# Time and Motion at the Endoscopy Unit—A University Hospital Experience

**DOI:** 10.1177/23333928231159808

**Published:** 2023-03-09

**Authors:** Simon Söderberg, Nils Nyhlin, Axelina Moro, Christina Figaro, Emelie Fransson, Jennie Stefansdotter, Malin Schagerström, Maria Lindblad, Martin Ahlzén, Olga Zukovets, Sofia Borell, Viktoria Johansson, Marianne Axman, Anette Wendt, Hanna Falck, Michiel A. van Nieuwenhoven

**Affiliations:** Department of Internal Medicine, Division of Gastroenterology, Faculty of Medicine and Health, Örebro University, Örebro, Sweden

**Keywords:** efficiency, gastroenterology, impact evaluation, mixed methods, practice management, endoscopy

## Abstract

**Background/aims:**

An effective workflow at the endoscopy unit is important for optimal production. We conducted a time-and-motion study to identify the amount of time that patients spend during the different steps of a regular endoscopy procedure and compared propofol with midazolam sedation.

**Methods:**

Data from 376 patients were prospectively collected. Durations of the different procedure steps were measured. Correlations between recovery times, age, and dose of sedative were calculated. Multiple regression analysis was performed to evaluate how various factors affect recovery time.

**Results:**

The use of midazolam resulted in significantly shorter procedure duration for gastroscopy (5.1 vs 8.3 min), shorter endoscopist delay duration for either types of endoscopy (5.9 vs 8.3 min for gastroscopy and 6.7 vs 11.4 min for colonoscopy), shorter endoscopy room duration for gastroscopy (22.2 vs 30.0 min), shorter recovery time for colonoscopy (23.4 vs 27.4 min) and shorter Endoscopy Unit Duration for either type of endoscopy (77.1 vs 101.4 min for gastroscopy and 99.6 vs 123.2 min for colonoscopy). There was a weak correlation between dose of midazolam and recovery time.

**Conclusions:**

In contrast to other studies, propofol administration leads to more time spent at different steps in the workflow at our unit. Implementing propofol sedation will not improve efficacy if other steps in the workflow are not taken into account.

## Introduction

An effective patient workflow at the endoscopy unit is mandatory for an effective production. The workflow is complex and there are various steps between the moment that the patient reports at the reception and the moment that the patient leaves the endoscopy unit. Identification and analysis of these steps makes it possible to identify bottlenecks and allows for adjustments to increase the efficacy of the workflow.

Midazolam, fentanyl, or other short-acting opioids and propofol are commonly used for sedation and/or pain relief worldwide. Although these drugs often successfully sedate, they result in short-term, reversible decline in cognitive function.^1,2^

Midazolam is a benzodiazepine traditionally used either alone or in combination with an opioid with sedation and reduction of discomfort and/or pain as goals. Midazolam is administered intravenously, and the dosage is adjusted to the patient's need and the duration of the procedure. The dosage varies considerably and is usually between 1.0 and 10 mg. Adverse effects, although not very common, comprise nausea and vomiting.^3,4^

Propofol is an anesthesia inducer with a rapid onset of action, anti-emetic effects, and a short half-life.^5,6^ The use of propofol, as an alternative to traditional sedation with midazolam and/or alfentanil when performing gastrointestinal endoscopy, has become increasingly popular in recent years around the world. Propofol sedation leads to faster onset of action, and is supposed to lead to faster recovery after endoscopy, and hence, faster discharge time and a higher patient satisfaction.^7–10^ A larger meta-analysis clearly shows an improvement in procedural volumes, better sedation, improved patient satisfaction, and shorter recovery times when propofol is used for routine endoscopy and colonoscopy.^11^ One systematic review article reported that propofol is even safer than midazolam, when administered under correct supervision, despite the complication risks associated with deep sedation.^5^

The administration of propofol and the continuous monitoring of vital signs during the endoscopy procedure requires more preparations before the endoscopic procedure, and there are usually 2 nurses present during the procedure. In contrast, sedation with midazolam, either alone or in combination with an opioid, usually requires only 1 nurse during the endoscopic procedure. The type of sedation depends on the procedure type, patient characteristics, the endoscopists’ earlier experience, and resource availability.^12^ There are studies that showed that patients with liver cirrhosis should receive sedation with propofol in order to reduce the risk of postprocedural encephalopathy.^10,13^

Annually, the endoscopy unit of the Örebro University Hospital performs approximately 6500 routine endoscopies: 3500 gastroscopies and 3000 colonoscopies. A minority of endoscopies consists of other procedures. The number of procedures increases every year, due to an ageing population and the implementation of colon cancer screening. In order to optimize the efficacy for this increasing demand, we decided to perform a time-and-motion study during the daily routine work at the endoscopy unit, in order to identify all the different steps that a patient undergoes between entering and leaving the endoscopy unit.

### Aim

We performed a real-life, observational study where we identified and measured the exact duration of every step of the endoscopy workflow and evaluated differences between midazolam and propofol sedation, for both gastroscopy and colonoscopy. In addition, we wanted to identify which type of sedation results in the shortest recovery time, which is important because a rapid discharge leads to a more efficient patient flow at the endoscopy unit.

## Methods

### Endoscopy Unit Structure and Workflow

The workflow analysis was performed at the endoscopy unit at the Örebro University Hospital. This unit is an outpatient unit, operating between 08:00 to 17:00 on weekdays, except Fridays, when the unit operates from 08:00 to 12:00. Different kinds of endoscopy procedures are performed at the endoscopy unit. This study, however, only focuses on routine gastroscopy and colonoscopy, since they constitute the vast majority of endoscopic procedures. The use of propofol is reserved for Mondays and Fridays, due to the limited availability of nurses. The unit has 4 endoscopy rooms. At least 3 rooms are used all weekdays. Endoscopists are assigned to 1 room for each session (either morning and/or afternoon).

The patient receives information with instructions as well as a calling time by mail or, if already admitted to the hospital, by the endoscopy unit staff. Patients who are called for an endoscopy procedure, first report at the reception, and thereafter directed to the waiting room to await for the procedure. Once at the unit, the patients are either directed to the dressing room in case of a colonoscopy, or to the preparation room or, if possible, directly to the endoscopy room. Subsequently, the patient receives an intravenous cannula and information about the procedure. Once an endoscopy room is available and prepared, the patient is moved to this room. When the patient is ready for the procedure, the endoscopist is notified by a nurse, enters the room, and starts with the procedure. After the procedure, the patient is moved to the recovery room in case midazolam or propofol was administered. If this was not the case, the patient is informed about the outcome of the endoscopy and can leave the endoscopy unit. In the recovery room, patients recover from the received sedative. Thereafter, the patient can leave the unit. When the endoscopy is finished and the patient has left the endoscopy room, it is cleaned and prepared for the next procedure.

A patient who is already admitted to the hospital does not have to report at the unit reception first, and is moved directly to the endoscopy unit corridor instead, where he/she waits for an available room. If necessary, the patient will receive an intravenous cannula. After the endoscopy, the patient returns to the endoscopy corridor where he/she waits to go back to the ward. These patients never enter the recovery room and hence, they were not included in the recovery time analysis.

### Study Population

We conducted this prospective cohort study during 31 nonconsecutive days between September and November 2021, in patients undergoing either a routine gastroscopy or colonoscopy with sedation at the Endoscopy Unit at the Örebro University Hospital. For sedation, the patients received either a bolus midazolam+/alfentanil or nurse-assisted propofol sedation consisting of approximately 50 mg bolus, plus additional propofol during the procedure to ensure adequate sedation. With colonoscopies, patients who received midazolam also received a bolus of alfentanil in almost all cases. The dosage of alfentanil that we used was 0.25 to 0.5 mg. In gastroscopies, we never use opioids. We did not use midazolam antidote. Opioids are never used in combination with propofol.

### Time Definitions

In order to evaluate the time spent during different steps at the endoscopy unit, we used the time definitions presented in Table 1.

**Table 1.** Explanation of the Used Time Definitions.

Workflow process measure	Definition
Calling time	Time patients received to announce their presence at unit reception
Reception registration time (*RegT*)	Actual time patients announce their presence at unit reception
Preparation room time (*PrepT*)	Time patients enter the preparation room
Entering endoscopy room time (*EnERT*)	Time patients enter the procedure room
Endoscopist room time (*EndoRT*)	Time endoscopist enters the procedure room
Procedure start time (*PStaT*)	Time of procedure start
Procedure stop time (*PStoT*)	Time of procedure stop
Procedure duration	Duration of procedure *PStaT – PStoT*
Exiting endoscopy room time (*ExERT*)	Time patients exit the procedure room
Recovery room time (*RecT*)	Time patients enter the recovery room
Leaving time (*LT*)	Time patients leave the endoscopy unit
Room turn-over time	The duration of time to prepare a procedure room for the next procedure (ExERT)−(Subsequent patient's EnERT in the same room)
Preparation room duration	Duration of time patients spend in the preparation room *EnERT – PrepT*
Endoscopy room duration	Duration of time patients spend in the procedure room *ExERT – EnERT*
Recovery time	Duration of time patients spend in the recovery room *LT – RecT*
Endoscopy unit duration	Duration of time patients spend at the endoscopy unit *LT – RegT*
Endoscopist delay duration	Duration of time between patients’ entry into the procedure room and endoscopists’ entry *EndoRT – EnERT*
	

### Data Collection

The time stamps used in this study were collected from either our computer system (Sectra Medical AB, Linköping, Sweden) or from direct observation. The time stamps collected from Sectra were calling time and reception registration time. All the other steps were measured via direct observation. There was only one observer and this was the same person during the whole study. The different time stamps were measured with a stopwatch. The different durations were then calculated. In addition, the dosages of sedation were registered.

### Analysis

All statistical analyses were performed using SPSS Statistics version 26 (IBM Corp., Armonk, NY, USA). Duration variables were analyzed for mean times and 95% confidence intervals. Statistical analysis was performed using the Mann–Whitney U test, as most duration variables were non-normally distributed. Bivariate correlation analysis was performed with either Pearson's test if variables were normally distributed, or Spearman's test if this was not the case. Multiple regression analysis was performed to see how age, sex, and dose of sedation affect variables. A power analysis was not performed since there are no previous data.

### Ethical Approval

The study was approved by the Swedish Ethical Review Authority with registration number 2019-00271.

## Results

Data were collected from 376 patients; 199 underwent a gastroscopy and 177 a colonoscopy. Of the 199 patients who underwent a gastroscopy, 172 (85 females) received midazolam, and the remaining 27 (13 females) received propofol. Of the 177 patients who underwent a colonoscopy, 154 (84 females) received midazolam and 23 (12 females) received propofol. The mean age of the 326 patients receiving midazolam was 63.1 years, and 45.8 years for the 50 patients receiving propofol.

The mean midazolam dosage for patients who underwent a gastroscopy was 2.7 mg. The most frequently used dose was 2.5 mg, and for colonoscopy the mean and most frequent dosages were 3.0 and 2.5 mg, respectively. The dosage of used propofol depended on the grade and need of individual sedation and showed a high intraindividual variation. Typically, the used dosages of propofol varied between 100 and 400 mg.

Table 2 shows the summary of results of the durations of all the steps and how they differ, depending on the used sedative.

**Table 2.** Durations of Different Variables in the Endoscopy Workflow, When Using Either Midazolam or Propofol.

Duration variable and groups	n	Median time (min)	Range
*Procedure duration*	376		
Gastro*			
Midazolam	172	5.1	22.8
Propofol	27	8.3	25.6
Colo			
Midazolam	154	25.5	98.3
Propofol	23	24.6	42.7
Either			
Midazolam	326	13.6	98.3
Propofol	50	13.7	18.8
*Endoscopist delay duration*	373		
Gastro*			
Midazolam	172	5.9	26.7
Propofol	27	8.3	21.7
Colo*			
Midazolam	154	6.7	35.5
Propofol	23	11.4	26.8
Either***			
Midazolam	326	6.4	35.5
Propofol	50	10.0	26.8
*Endoscopy room duration*	376		
Gastro***			
Midazolam	172	22.2	35.2
Propofol	27	30.0	53.9
Colo			
Midazolam	154	44.1	107.3
Propofol	23	47.5	75.5
Either**			
Midazolam	326	30.8	121.3
Propofol	50	36.9	83.6
*Recovery time*	285		
Gastro			
Midazolam	115	32.9	111.1
Propofol	22	33.4	70.2
Colo*			
Midazolam	126	23.4	91.4
Propofol	22	27.4	95.1
Either			
Midazolam	241	26.6	111.7
Propofol	44	29.2	97.5
*Endoscopy unit duration*	355		
Gastro*			
Midazolam	159	77.1	199.6
Propofol	25	101.4	107.1
Colo*			
Midazolam	149	99.6	205.9
Propofol	22	125.2	150.4
Either**			
Midazolam	308	91.5	231.3
Propofol	47	110.9	190.6

The asterix (*) indicates degree of statistical significance: * means p ≤ 0.05, ** means p ≤ 0.01, and *** means p ≤ 0.001. Statistical analysis was performed using Mann–Whitney U test.

Abbreviations: Colo, colonoscopy; Gastro, gastroscopy.

### Age and Its Effect on Outcomes

Neither age nor the recovery time of these patients were normally distributed, as shown by the Shapiro–Wilk's test (p > 0.05). Spearman's analysis showed no correlation between age and recovery time for the patients receiving either midazolam or propofol who underwent either gastroscopy or colonoscopy (ρ=−0.011, p = 0.857), and no correlation between age and endoscopy unit duration (ρ=−0.82, p = 0.122).

### Dose of Sedation and Recovery Time

The dose of sedation given before and during the endoscopic procedure may affect the amount of time the patients spend recovering and being present at the unit overall. The 226 patients eligible for this subanalysis underwent either a gastroscopy or colonoscopy and were divided into 6 unequal groups depending on the midazolam dose (1.25, 2.5, 3.75, 5, 6.25, or 7.5mg). Spearman's analysis was performed to assess correlation. There was a significant, weak positive correlation between the dose of midazolam and recovery time (ρ=0.287, p < 0.001). For propofol, there was no correlation between the dose and the recovery time (ρ=0.198, p = 0.227).

### Dose of Sedation and Endoscopy Unit Duration

Spearman's correlation analysis was performed for both gastroscopy and colonoscopy. There was a significant, moderately strong positive correlation between the doses of midazolam and endoscopy unit duration in the gastroscopy group (n = 159) (ρ=0.334, p < 0.001) and a significant, weak positive correlation in the colonoscopy group (n = 149) (ρ=0.229, p = 0.005). For propofol, the patients were divided into gastroscopy and colonoscopy groups, since the endoscopic procedure probably affects the endoscopy unit duration more than the recovery time does. Pearson's analysis showed no correlation between the dose of propofol and endoscopy unit duration for both the gastroscopy (n = 26) (R = 0.307, p = 0.127) and the colonoscopy groups (n = 27) (R = 0.252, p = 0.245).

### Regression Analysis

We performed multiple regression analysis to analyze how the factors sex, age, and the dose of midazolam could predict recovery time. Recovery times and the independent variables were collected from 241 patients who underwent either gastroscopy or colonoscopy.

Sex, age, and dose of midazolam significantly accounted for 11.9% of the variance in recovery time with an adjusted R^2^ of 10.8% and significantly predicted recovery time (F(3, 237) = 10.67, p < 0.001, R^2 ^= 0.119, adjusted R^2 ^= 0.108). Testing these independent variables (the coefficients) separately for the variance (with α=0.05) using analysis of variance, dosage of midazolam and age showed significance (p < 0.001 and p = 0.004, respectively), while sex did not (p = 0.157).

The overall model for sex, age, and dose of propofol was not statistically significant (F(3,40) = 0.367, p = 0.778, R^2 ^= 0.027, adjusted R^2^=−0.046).

### Room Turn-Over Times

We also measured room turn-over time. In total, data from 265 patients were collected. The mean room turn-over time was 13.0 min. There were no significant differences between midazolam and propofol sedation or between gastroscopy and colonoscopy.

## Discussion

This study shows that a time-and-motion study can be used to identify the durations of all the different steps of an endoscopy procedure. At our unit, we found that for most duration variables, propofol administration led to increased time spent at the endoscopy unit in most cases.

The findings from our study differ from the findings of other studies. Lazzaroni et al^14^ concluded that propofol administration resulted in a shorter time until full recovery was reached and that discharge times were shorter. Another study^15^ concluded that propofol, when compared to a combination of midazolam/fentanyl/propofol and midazolam/fentanyl, reduced procedure time for gastroscopies and both recovery and procedure time for colonoscopies. Koshy et al^16^ reported that propofol plus fentanyl compared to midazolam plus meperidine, led to shorter recovery times, although nonsignificantly. There are several other studies that showed that the use of propofol shortens the recovery time, and consequently, discharge time of patients undergoing an endoscopic procedure.^5,6,17^

We identified a number of possible explanations for the discrepancy between our results and the existing literature. Firstly, patients who received propofol were more often asleep when entering the recovery room, compared to patients who received midazolam, and therefore they received instructions after a nurse had taken notice of their awakening, which affects recovery time

Secondly, in-hospital patients awaited in the unit corridor before shifting to their ward. Their departure depended on the arrival of transportation staff, and not by the endoscopy unit staff. These patients were not excluded from the analysis of endoscopy unit duration and its correlations, but since they did not enter the observation room, they were not included in the analysis of recovery time.

Thirdly, as a routine procedure, all patients who underwent a colonoscopy with midazolam, also received the opioid alfentanil. This was not the case with propofol sedation. Alfentanil, and opioids in general, also affect the state of arousal and cognitive function,^18^ which, in turn, also affects the time to full recovery. The interpretation of presented results and the following conclusions should therefore take the use of opioids into account.

We used the variable “recovery time” as a way to assess the patients’ recovery ability. However, it is unsure whether this accurately reflects this ability, since patients may be adequately recovered, but are waiting for the nurse to get permission to leave the unit. At our hospital, we use bolus propofol plus additional propofol, if needed. There are studies^19–21^ that demonstrate the efficacy of continuous infusion propofol for selected endoscopy and colonoscopy procedures in terms of efficacy and decrease of recovery time. Finally, there are other variables that may affect recovery time, but also procedure duration, such as comorbidities, other medications, kidney function, liver function, body weight, smoking, etc. Hence, we cannot exclude a certain selection bias regarding the choice for midazolam or propofol, based on these types of variables.

There are a number of other factors that could affect the durations of the different steps, specifically regarding procedure duration, recovery time, and endoscopy unit duration. These factors include the experience of the endoscopist and other staff; the teaching of a student/resident; whether or not the unit was fully staffed, and the complexity of the procedure. Although these aspects exist, they are assumed to be equally distributed between both groups.

Some variables, namely endoscopist delay duration, endoscopy room duration, and room turn-over times, are interesting variables for reflection about the overall patient workflow. For instance, this study shows that the endoscopy delay durations were larger in the propofol group for both endoscopic procedures. This is not surprising, since preparing a patient for propofol sedation takes more time, compared to midazolam. The practical implication may be that, in case of propofol, the endoscopist can be scheduled to other tasks.

Another relevant variable is room turn-over time. Optimal logistics at the work floor may shorten this turn-over time and improve the workflow efficacy.

Although the strength of our study includes the fact that it mirrors the daily routine workflow at an endoscopy unit, it inevitably has its weaknesses. In total, we included 50 patients who received propofol and 326 patients who received midazolam. The reason for this difference is that midazolam is more frequently used at our unit due to logistical reasons, such as the availability of 2 nurses in the endoscopy room. When dividing each sedation group into gastroscopy and colonoscopy, the population sizes reduced to approximately half, resulting in data that may not have sufficient power to draw definitive conclusions. Finally, since this is a real-life observational study, the patients were not randomized to receive either midazolam or propofol, leading to selection bias, as reflected in the age difference between the study groups. The choice for the type of sedation was made by the gastroenterologist who assessed the referrals, and was mainly based on parameters such as patient anxiety, the preference of the patient or on the suggestion of the referring doctor, usually a general physician. On the other hand, the groups were not selected based on medical history including drugs, and body mass index, as long as it was under 40 kg/m^2^, which was the case in our study population, or the need for biopsies or polypectomy. Endoscopic mucosal resection/endoscopic submucosal dissection procedures were not performed at all in this study population.

We conclude that a time-and-motion study is very useful to identify the durations of all the different steps of an endoscopy procedure, thus allowing for the identification of potential bottlenecks. At our unit, we observed that the administration of propofol leads to an increased endoscopist delay duration for both gastroscopy and colonoscopy, an increased procedure duration in case of a gastroscopy, an increased endoscopy room duration in case of a gastroscopy, an increased recovery time in case of a colonoscopy and an increased endoscopy unit duration for both gastroscopy and colonoscopy. The age of the patients did not affect recovery time or endoscopy unit duration.

In contrast to other studies, we were unable to confirm that propofol sedation leads to a faster discharge of the patient. The use of continuous propofol administration may improve this aspect. The dose of administered midazolam correlates positively with the time spent in the recovery room for patients, as well as the total time at the unit for patients undergoing an endoscopy. The use of midazolam antidote may shorten this time. We conclude that implementation of propofol with the aim to increase the efficacy, is not sufficient when other steps of the endoscopy workflow are not taken into account. We will use these data to improve the efficacy of the workflow at our unit.
